# Two Days Versus Five Days of Postoperative Antibiotics for Complex Appendicitis: Cost Analysis of a Randomized, Noninferiority Trial

**DOI:** 10.1097/SLA.0000000000006089

**Published:** 2023-09-12

**Authors:** Elisabeth M.L. de Wijkerslooth, Evert-Jan G. Boerma, Charles C. van Rossem, Marc A. Koopmanschap, Coen I.M. Baeten, Frédérique H. Beverdam, Joanna W.A.M. Bosmans, Esther C.J. Consten, Jan Willem T. Dekker, Marloes Emous, Anna A.W. van Geloven, Anton F. Gijsen, Luc A. Heijnen, An P. Jairam, Augustinus P.T. van der Ploeg, Pascal Steenvoorde, Boudewijn R. Toorenvliet, Maarten Vermaas, Bas Wiering, Bas P.L. Wijnhoven, Anne Loes van den Boom

**Affiliations:** * Departments of Surgery, Erasmus MC—University Medical Center, Rotterdam, The Netherlands; † Departments of Surgery, Zuyderland Medical Center, Heerlen/Sittard, The Netherlands; ‡ Departments of Surgery, Maasstad Hospital, Rotterdam, The Netherlands; § Departments of Surgery, Erasmus School of Health Policy and Management, Erasmus University, Rotterdam, The Netherlands; ∥ Departments of Surgery, Groene Hart Hospital, Gouda, The Netherlands; ¶ Departments of Surgery, Franciscus Gasthuis and Vlietland, Rotterdam, The Netherlands; # Departments of Surgery, Meander Medical Center, Amersfoort, The Netherlands; ** Departments of Surgery, University Medical Center Groningen, The Netherlands; †† Departments of Surgery, Reinier de Graaf Gasthuis, Delft, The Netherlands; ‡‡ Departments of Surgery, Medical Center Leeuwarden, Leeuwarden, The Netherlands; §§ Departments of Surgery, Tergooi Medical Center, Hilversum/Blaricum, The Netherlands; ∥∥ Departments of Surgery, Medical Spectrum Twente, Enschede, The Netherlands; ¶¶ Departments of Surgery, Northwest Clinics, Alkmaar/Den Helder, The Netherlands; ## Departments of Surgery, Catharina Hospital, Eindhoven, The Netherlands; *** Departments of Surgery, Ikazia Hospital, Rotterdam, The Netherlands; ††† Departments of Surgery, IJsselland Hospital, Capelle a/d Ijssel, The Netherlands; ‡‡‡ Departments of Surgery, Slingeland Hospital, Doetinchem, The Netherlands

**Keywords:** antibiotics, appendicitis, costs, cost-effectiveness

## Abstract

**Objective:**

To compare costs for 2 days versus 5 days of postoperative antibiotics within the antibiotics after an aPPendectomy In Complex appendicitis trial.

Background:

Recent studies suggest that restrictive antibiotic use leads to a significant reduction in hospital stays without compromising patient safety. Its potential effect on societal costs remains underexplored.

**Methods:**

This was a pragmatic, open-label, multicenter clinical trial powered for noninferiority. Patients with complex appendicitis (age ≥ 8 years) were randomly allocated to 2 days or 5 days of intravenous antibiotics after appendectomy. Patient inclusion lasted from June 2017 to June 2021 in 15 Dutch hospitals. The final follow-up was on September 1, 2021. The primary trial endpoint was a composite endpoint of infectious complications and mortality within 90 days. In the present study, the main outcome measures were overall societal costs (comprising direct health care costs and costs related to productivity loss) and cost-effectiveness. Direct health care costs were recorded based on data in the electronic patient files, complemented by a telephone follow-up at 90 days. In addition, data on loss of productivity were acquired through the validated Productivity Cost Questionnaire at 4 weeks after surgery. Cost estimates were based on prices for the year 2019.

**Results:**

In total, 1005 patients were evaluated in the “intention-to-treat” analysis: 502 patients were allocated to the 2-day group and 503 to the 5-day group. The mean difference in overall societal costs was – €625 (95% CI: –€ 958 to –€ 278) to the advantage of the 2-day group. This difference was largely explained by reduced hospital stay. Productivity losses were similar between the study groups. Restricting postoperative antibiotics to 2 days was cost-effective, with estimated cost savings of €31,117 per additional infectious complication.

**Conclusions:**

Two days of postoperative antibiotics for complex appendicitis results in a statistically significant and relevant cost reduction, as compared with 5 days. Findings apply to laparoscopic appendectomy in a well-resourced health care setting.

Acute appendicitis is a common disease among children and adults.^1^ Although nonoperative management is gaining popularity,^2^ appendectomy is still performed in most patients. Complex appendicitis (appendicitis with necrosis, perforation, or abscess) is seen in ~30% of patients who undergo appendectomy. In these patients, postoperative antibiotics are given in an attempt to reduce infectious complications.^3–6^ The duration of postoperative antibiotics remains under debate.^7–12^ Benefits of restrictive antibiotic use include reduced hospitalization and reduced adverse effects related to antibiotics. The growing issue of antimicrobial resistance (AMR) calls for antibiotic stewardship.^13–15^

In international literature on acute appendicitis, a gap in the literature was acknowledged considering the optimum duration of antibiotic use for complex appendicitis.^16–19^ The antibiotics after an aPPendectomy In Complex (APPIC) appendicitis trial confirmed what previous studies^11,12,18–20^ suggested: 2 days of intravenous (iv) antibiotics was comparably effective and safe compared with 5 days.^21^ Whereas safety and efficacy are key components in determining treatment strategy, costs, and cost-effectiveness should also be considered. Health care costs have risen over the past decades in both developed and developing countries. The coronavirus disease 2019 pandemic has further accelerated this. The latest WHO report on global health expenditure showed that health care spending keeps rising across developed and developing countries.^22^ In many high-income countries, health care expenditure has surpassed 10% of the gross domestic product (data available online in the Global Health Observatory repository by the WHO).^23^ This calls for a critical review of cost-effectiveness in all fields of medicine to identify opportunities to cut costs. As the annual number of patients treated for acute appendicitis is high, a reduction in direct- or indirect costs can have a large effect.

We hypothesized that direct health care costs would be lower for patients in the 2-day group but that productivity costs would be similar, as efficacy outcomes in both trial groups were comparable.

The aim of this study was to compare overall societal costs and evaluate cost-effectiveness for 2 days versus 5 days of postoperative iv antibiotics for complex appendicitis.

## METHODS

### Trial Design and Oversight

The APPIC trial was a pragmatic, nonblinded, randomized controlled trial powered for noninferiority. The trial design was registered in the Netherlands Trial Register in December 2016 (code NL5946) and was published in May 2018.^24^ The trial was approved by the ethics committees at all participating sites (1 academic center and 14 teaching hospitals in the Netherlands). Details of the trial design and methods were published.^21,24^ This manuscript was prepared in accordance with the “Consolidated Standards of Reporting Trials” checklist for noninferiority randomized clinical trials and the Consolidated Health Economic Evaluation Reporting Standards checklist for economic evaluations of health interventions.^25,26^

### Patients

Patients aged ≥8 years were enrolled in the study if they underwent an appendectomy for complex appendicitis and gave informed consent. Complex appendicitis was defined as the presence of necrosis, perforation, or abscess, as assessed intraoperatively.^3^ Patients were excluded from participation if they were pregnant, immunocompromised, or American Society of Anesthesiologists class IV. Other exclusion criteria are provided in the full protocol.^21^ All patients gave written informed consent.

### Randomization

Within 24 hours after the appendectomy trial, patients were randomized to 2 days or 5 days of postoperative antibiotics in a 1:1 ratio. Computerized block randomization was used (random-sized blocks, size range: 4–8), stratified for the center. Treating physicians and patients were not blinded to treatment allocation.

### Treatment

The postoperative antibiotic regimen consisted of cefuroxime (1500 mg three times daily) or ceftriaxone (2000 mg once daily) in combination with metronidazole (500 mg three times daily), administered intravenously. In children, the dosage was adjusted according to their weight. A daily single dose of gentamycin iv was allowed as cointervention. After 2 days or 5 days, antibiotics were ceased. The following deviations in regimen were allowed: (1) switch in an antibiotic agent and/or prolongation due to perioperative culture results, (2) early discontinuation due to adverse effects (ie, allergic reaction and thrombophlebitis) or repeated iv failure, and (3) switch in an antibiotic agent and/or prolongation due to an infectious complication.

### Procedures

Diagnostic work-up, preoperative antibiotic prophylaxis, and surgical approach were according to local hospital standards. Postoperative laboratory tests, imaging studies, and blood cultures were performed upon clinical indication, according to local protocol. Final discharge, as well as type and timing of follow-up, were left to the discretion of the treating physician. Four weeks after an appendectomy, patients received a Productivity Cost Questionnaire (PCQ) by mail. If unanswered, they received another. Follow-up lasted until 90 days after appendectomy, upon which a telephone follow-up was conducted by the central trial coordinator. Data acquired from the electronic patient files, the questionnaire, and the telephone follow-up were registered in a secure online database in a pseudonymized manner.

### Outcomes

The primary endpoint of the trial was a composite endpoint of infectious complications and mortality within 90 days after appendectomy. Infectious complications included intra-abdominal abscess and surgical site infections.^27^ Secondary outcomes included postoperative antibiotic use, overall postoperative complications, reinterventions, hospital readmission, length of hospital stay, and visits to the emergency room, outpatient clinic, or general practitioner. All within 90 days after surgery.

The primary outcome presented in this study is the estimate of overall societal costs for both treatment allocations. Overall societal costs are the sum of direct health care costs and estimated costs due to loss of productivity. The secondary outcome is the cost-effectiveness of the 2-day versus 5-day treatment allocation.

### Statistical Analysis

Details of the sample size calculation and statistical analysis of primary and secondary trial endpoints were published.^21,24^ Overall societal costs were estimated for each patient included in the “intention-to-treat” analysis, from the moment of surgery until 90 days thereafter. Direct health care costs included the cost of surgery, antibiotic use (iv and oral), inpatient days (including intensive care unit stay and readmission stay), imaging studies, reinterventions, and visits to the emergency room, outpatient clinic, and general practitioner. Costs per unit were based on tariffs for surgery (Dutch Healthcare Authority, 2019), reference prices for health care costs (National Healthcare Institute of The Netherlands, 2015), and published prices of medication (www.medicijnkosten.nl, 2019).^28^

Costs due to loss of productivity entailed absence from paid work, lower productivity at work, and less unpaid work performed for patients aged ≥18. Cost estimates were derived from the validated PCQ,^29^ completed by patients 4 weeks after surgery. Data regarding absence from school or studies were also collected. In patients who did not complete or return the PCQ, productivity costs were estimated using a multivariate regression analysis on the data from patients who did return a completed PCQ. Factors included in the regression analysis were age, sex, length of hospital stay, and readmission (predictors for loss of productivity). Separate regression analyses were conducted using the data from patients aged 16 to 64 to estimate absence from paid work and reduced productivity, and data from patients aged ≥16 to estimate loss of unpaid work. For patients aged 8 to 15, multivariate regression analysis (including the same variables) was performed to estimate days of absence from school.

Cost-effectiveness was determined through the calculation of the incremental health care costs and productivity costs per additional/avoided infectious complication of 2 days versus 5 days of postoperative antibiotics. As “health effect” the primary endpoint of the study was used: occurrence of intraabdominal abscess, surgical site infection, and/or mortality within 90 days after appendectomy. The degree of uncertainty for both costs, health effects, and the cost-effectiveness ratio is depicted in a cost-effectiveness plane, based on nonparametric bootstrapping. Analyses were performed using SPSS (Version 25: IBM Corp) and Excel 2010 (Microsoft). Alpha level <0.05 was considered statistically significant.

## RESULTS

**FIGURE 1:**
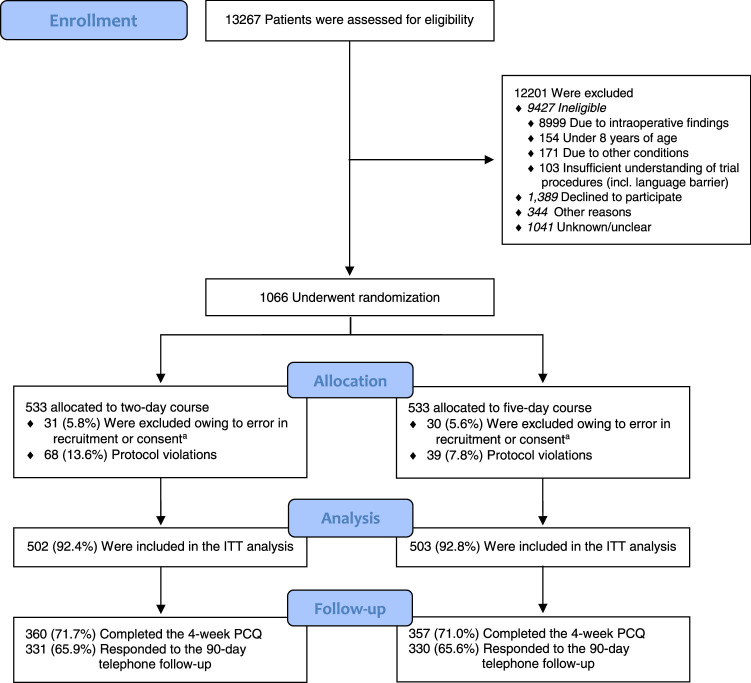
Screening, randomization, and follow-up. Subscript: “a” indicates 61 patients were excluded from analysis owing to an error in recruitment or consent: 14 due to a missed exclusion criterion (11 immunocompromised patients, 2 ASA IV patients, and 1 with a concurrent other indication for postoperative antibiotics), 39 due to incomplete/unsaved written consent and 8 due to patient withdrawal shortly after randomization. ASA indicates American Society of Anesthesiologists.

From June 2017 to June 2021, 1066 patients were randomized and 1005 were evaluated in the “intention-to-treat” analysis: 502 were allocated to the 2-day group and 503 to the 5-day group (Fig. 1). Results for the primary endpoint showed that 2 days of postoperative antibiotics was noninferior to 5 days, as infectious complications and mortality occurred in a similar proportion of patients (10.2% vs 8.2%, respectively; absolute risk difference 2.0%, 95% CI: −1.6 to 5.6).^23^ The mean ± SD total length of hospital stay was 4.4 ± 3.3 days in the 2-day group, compared with 6.1 ± 3.0 days in the 5-day group (*P* < 0.001).

The PCQ was completed and returned by 717/1005 patients (71%), 360 in the 2-day group and 357 in the 5-day group. The telephone follow-up was answered by 664/1005 patients (66%), 330 in the 2-day group and 331 in the 5-day group. The patient-reported data was insufficient for analysis in 8 patients, leaving 709/1005 patients (71%) with complete data for analysis. For the remaining 296 patients, productivity losses were calculated through multivariable regression analysis.

### Societal Costs

Overall societal costs per patient were €625 lower in the 2-day group compared with the 5-day group (95% CI: –€ 958 to –€278). Table 1 shows the details of direct health care costs and productivity losses. Direct health care costs were €704 (95% CI: –€ 932 to –€ 482) lower in the 2-day group compared with the 5-day group, mainly as a result of a reduced length of stay. The difference in productivity losses was not statistically significant. The mean number of missed school days per student was similar in both arms: 9.0 ± 5.9 in the 2-day group versus 9.4 ± 5.0 in the 5-day group.

**TABLE 1:** Overall Societal Costs for the Intention-to-Treat Population (N = 1005)

	2-day Arm (N= 502)	5-day Arm (N = 503)
Direct health care costs (€)	3922 (1912)	4626 (1619)
Surgery^*^	1648	1641
Postoperative antibiotic use	76	131
Postoperative imaging studies	193	184
Inpatient days^†^	1939	2627
ER and outpatient visits	62	39
General practitioner visits	5	4
Productivity losses (€)	2223 (2085)	2145 (2032)
Absence paid work	1772	1698
Lower productivity paid work	155	155
Unpaid work	297	291
Overall societal costs (€)	6146 (2891)	6771 (2607)

Data are presented as means or means (SD).

* Includes reoperation.

† Includes readmission.

ER indicates emergency room.

### Cost-effectiveness

**FIGURE 2:**
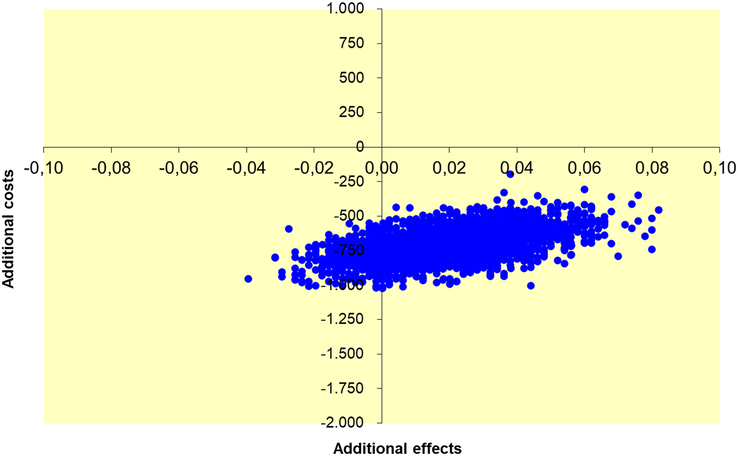
Uncertainty plot for 2 days versus 5 days of postoperative antibiotics (health care perspective).

**FIGURE 3:**
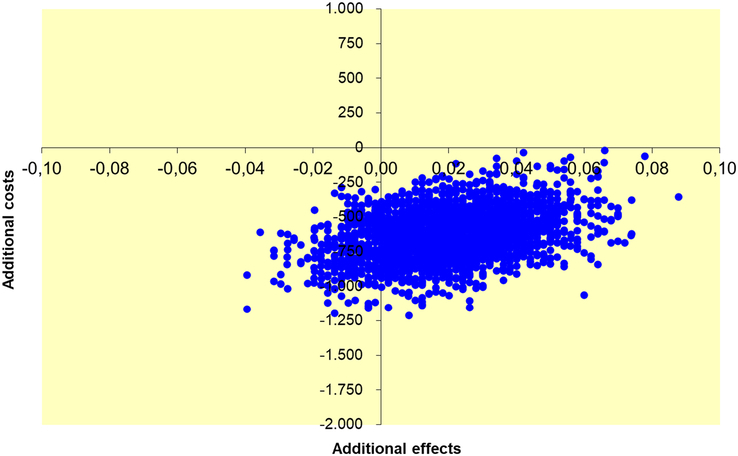
Uncertainty plot for 2 days versus 5 days of postoperative antibiotics (societal perspective).

The absolute risk difference in the primary endpoint, used as the effectiveness parameter, was −2.0% (95% CI: −1.56% to 5.57%).^23^ Cost-effectiveness analysis demonstrates €31,117 savings in overall societal costs per additional infectious complication as a result of 2 days versus 5 days treatment allocation (Table 2). Taking into account only direct health care costs, savings were €35,032 per additional infectious complication in the 2-day group. Figures 2 and 3 show a 100% probability that switching from 5 days to 2 days of postoperative antibiotics saves direct health care costs and societal costs. The probability of additional complications for 2-day treatment is 87%, whereas the chance of fewer complications is 13%.

**TABLE 2:** Cost-effectiveness of 2 Days Versus 5 Days of Postoperative Antibiotics

	Health care system perspective^*^	Societal perspective^*^
Incremental cost	−€704 (−932 to −482)	−€ 625 (−958 to −278)
Additional fraction complications	+0.0201	+0.0201
Incremental cost per extra complication	−€ 35,032	−€ 31,117

Incremental cost is presented as the mean difference (95% CI).

* The health care system perspective is based solely on direct health care costs, whereas the societal perspective also takes into account productivity losses.

## DISCUSSION

This is the first randomized study on postoperative antibiotic use for complex appendicitis to evaluate costs and cost-effectiveness. Overall societal costs were €625 lower in patients allocated to 2 days of antibiotics, representing a 9% cost reduction compared with patients allocated to 5 days. This difference mainly derives from shorter hospitalization in the 2-day group, whereas productivity losses were similar in both arms. Restricting postoperative antibiotics to 2 days was also demonstrated to be cost-effective, with estimated cost savings of €31,117 per additional infectious complication. These results consolidate the advantageous effect of restrictive antibiotic use for this indication, after the previous establishment of noninferiority in the prevention of postoperative infectious complications.^21^

During trial inclusion, 3 days of iv antibiotics (without oral antibiotics at discharge) has become more common in Dutch hospitals, in pursuit of the prospective cohort study by Van Rossem et al^8^ comparing 3 with 5 days of antibiotics in 2016. Nonetheless, recent data from our multicenter cohort study, including over 1500 patients with complex appendicitis, however, showed a mean duration of postoperative antibiotics of 4.8 days (iv and oral use together).^30^ Hopefully, the APPIC trial encourages surgeons to use a 2-day iv regimen as a standard of care. Its implementation is expected to lead to a substantial reduction in antibiotic use and hospital admission days, not only on a national level but also internationally.^21^ Hence, we make a plea for incorporating our data in guidelines, such as those by the World Society of Emergency Surgery and the Surgical Infection Society.^9,10^

Considering the growing issue of AMR, the present results may stimulate antibiotic stewardship.^14,20^ Ultimately, reducing antibiotic overuse for various indications, including appendicitis, may altogether slow down the emergence of AMR. AMR represents a silent pandemic associated with substantial mortality worldwide.^15^ In addition, AMR was shown to significantly correlate to prolonged hospitalization and increased hospital costs.^31^ Therefore, in the long term, total societal cost savings related to reduced antibiotics may be higher than presented in this paper.

Cost savings in this study were mainly related to reduced hospital stays, whereas there was no significant difference in costs related to productivity losses (ie, sick leave). The National Healthcare Institute in the Netherlands estimated that one day of hospital admission at a surgical department costs €405 on average, excluding diagnostics and medication.^28^ Studies also showed that laparoscopic surgery is associated with reduced hospital stay and sick leave, as compared with open surgery.^32,33^ Important to note is the high rate (97%) of laparoscopic procedures in the present trial for both arms. In populations with a higher rate of open appendectomy, postoperative hospital stay, sick leave, and related costs may be higher than reported in this study. Previous trials evaluating postoperative antibiotics for complex appendicitis have not presented data on costs.^7,11,12,34,35^ All studies demonstrated that restriction of antibiotics results in reduced length of hospital stay without a compromise in infectious complications or reinterventions. It could be speculated that this may have led to cost savings, as well in these studies.

The absolute costs presented in this study are difficult to compare to other studies assessing direct health care costs and productivity costs related to appendicitis, as different cost components are measured per study. Moreover, some studies report hospital charges,^36^ whereas others present the direct cost of resources, and the scale of cost savings may differ per country and region. Nevertheless, we found a 100% probability that reducing antibiotics for complex appendicitis will result in a significant reduction of related costs.

This study has some limitations. As described, there was a substantial loss of follow-up with 71% of productivity questionnaires returned and 66% of telephone follow-up answered. Patient characteristics related to loss of follow-up could be associated with hospital stay and sick leave. Though multivariable regression analysis was used to compute missing data, this may have delivered less accurate estimates of productivity costs in our study. Also, many factors contributing to direct health care costs were taken into consideration, but some components of the total costs may have been left unexplored. Apart from the surgical approach and postoperative antibiotic regimen, doctors’ recommendations may also have influenced sick leave. Patients discharged early in the 2-day group might have been advised differently than patients in the 5-day group. Thirdly, as mentioned, the rate of laparoscopic surgery was exceptionally high, which limits the generalizability of the outcomes in low to middle-income countries. Presented results are applicable to patients in a well-resourced health care setting. A strong element of this study was the large number of patients and the combined assessment of direct health care costs and productivity costs. This enabled an overall societal perspective of costs.

## CONCLUSIONS

This trial demonstrated that 2 days of postoperative iv antibiotics for complex appendicitis offers statistically significant savings in overall societal costs, as compared with 5 days. These savings derive mainly from reduced hospital admissions. Developing strategies to further restrict postoperative antibiotic use and minimize the length of hospital stay may deliver a substantial reduction in health care costs in the future.
